# In Every Man, There Is a Child: Henoch-Schönlein Purpura in an Adult With Liver Cirrhosis

**DOI:** 10.7759/cureus.18270

**Published:** 2021-09-25

**Authors:** Jiajia Yang, Andrew Okpe, Amogh Pathak

**Affiliations:** 1 Molecular Pharmacology and Physiology, University of South Florida Morsani College of Medicine, Tampa, USA; 2 Internal Medicine, St. Peter’s University Hospital, New Brunswick, USA

**Keywords:** henoch-schönlein purpura (iga vasculitis), glomerulonephritis, iga nephropathy, liver cirrhosis, alcohol use disorder

## Abstract

Henoch-Schönlein purpura (HSP), also known as immunoglobulin A (IgA) vasculitis, is a small-vessel vasculitis characterized by IgA deposits in various organs in the body producing a unique constellation of symptoms. This disease predominantly affects the skin (palpable purpura), joints (arthritis/arthralgia), gut (abdominal pain), and kidneys (nephritic syndrome-IgA nephropathy [IgAN]). The pathogenesis of HSP in children is usually secondary to an immune reaction after viral infections. In adults, few cases of HSP/IgA vasculitis have been reported secondary to altered metabolism of IgA in patients with alcoholic liver cirrhosis.

Here, we report an unusual case of HSP/IgA vasculitis. The patient presented with signs of alcoholic liver cirrhosis with abdominal pain and ascites along with a lower extremity purpuric rash. The patient had significant findings of liver cirrhosis with radiographic evidence of cirrhotic liver with esophageal varices and splenorenal shunt and elevated serum ascites albumin gradient. Urinalysis revealed proteinuria with microscopic hematuria, further evaluated with a kidney biopsy. Microscopic analysis revealed focal segmental endocapillary and extracapillary proliferative glomerulonephritis with focal necrotizing features, consistent with IgAN/HSP nephritis. Treatment was initiated with high-dose steroids and cyclophosphamide infusions.

Alcohol-induced endotoxin release and inflammation lead to high amounts of circulating IgA due to increased intestinal permeability and reduced hepatic clearance. Further disease development is caused by IgA deposits in affected organs (skin and kidney in our case). We hypothesize that the development of disease for the patient was secondary to altered IgA processing in decompensated alcoholic cirrhosis.

## Introduction

Henoch-Schönlein purpura (HSP, also known as immunoglobulin A [IgA] vasculitis) often involves the skin, gastrointestinal tract, kidney, and frequently causes arthritis [1]. HSP is primarily a childhood disease, affecting children between the ages of three and fifteen years, with a male predominance [2]. In adults presenting with HSP, intussusception is rarely reported, but the risk of progression of renal involvement to end-stage kidney disease is higher.

HSP nephritis may appear indistinguishable from immunoglobulin A nephropathy (IgAN) [1]. IgAN is characterized by the presence of IgA dominant or codominant mesangial deposits and is the most prevalent primary glomerulonephritis worldwide. Clinically, IgAN manifests with hematuria/proteinuria and impaired kidney function. It is classified as the primary idiopathic form and the secondary form that is associated with chronic liver disease (the leading secondary cause), chronic infections, and neoplasms [3]. Cirrhosis has been commonly implicated with IgA mesangial deposits with fewer other immunoglobulins and C3 in the glomeruli. The term cirrhotic glomerulonephritis is used to describe this pathological phenomenon [4].

## Case presentation

A 56-year-old Caucasian male with a history of alcohol use disorder presented to the hospital complaining of abdominal distention and moderately severe nonlocalizing aching pain all over the abdomen for one week and lower extremity petechial rash for four days.

**Figure 1:**
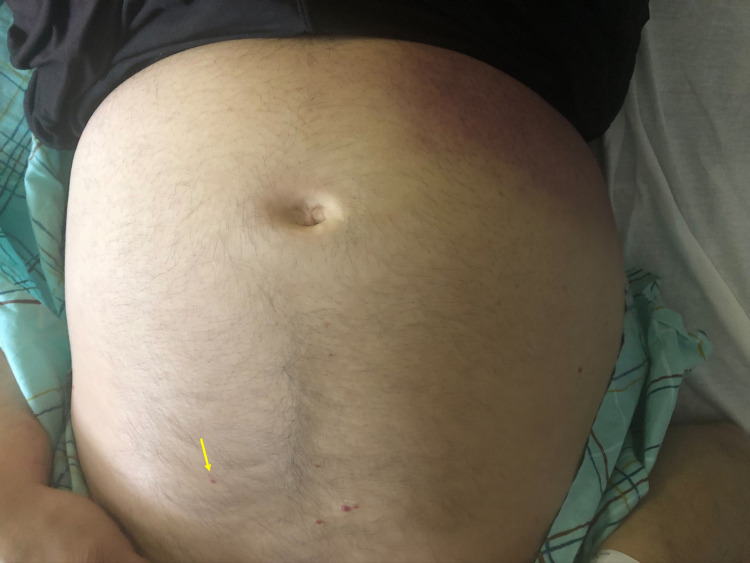
Abdominal distension with the petechial rash on the skin.

**Figure 2:**
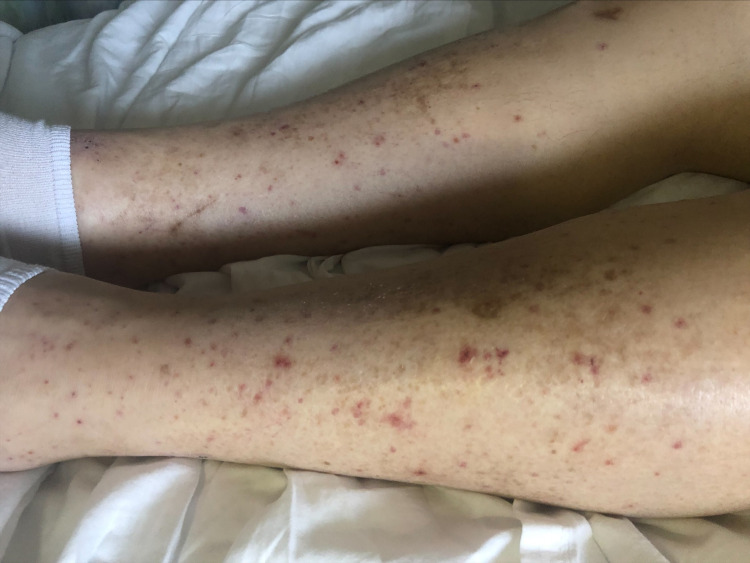
Bilateral lower extremity petechial rash.

Physical examination showed globular distention of the abdomen (Figure 1) with dullness to percussion in bilateral flanks with shifting dullness. In addition, he had an abdominal wall ventral hernia extending from the xiphisternum to the umbilicus. Examination of the skin also showed flat, nonblanching, pinpoint, round, reddish-brown petechial rash in clusters on bilateral shins (Figure 2) and abdomen (Figure 1).

The patient underwent computed tomography (CT) scan of the abdomen and paracentesis. CT scan revealed cirrhotic liver with ascites, extensive esophageal varices, and splenorenal shunt. Ultrasound further revealed signs of portal hypertension with splenomegaly. Diagnostic and therapeutic paracentesis was performed, with fluid studies nonindicative of spontaneous bacterial peritonitis. The albumin level of ascitic fluid was 0.6 g/dL, with a serum ascites albumin gradient of 1.9 g/dL.

Routine complete blood count was significant for hemoglobin of 13.6 g/dL with macrocytosis (mean corpuscular volume of 100.9 μm^3^) and thrombocytopenia (77,000/mm^3^). Effects of liver cirrhosis were apparent with the international normalized ratio of 1.26, low albumin (2.5 g/dL), and bilirubin of 3.9 mg/dL (with an indirect component of 2.6 mg/dL). Liver transaminases were within normal limits, and alkaline phosphatase was elevated at 131 U/L.

The patient had reduced kidney function with a creatinine of 1.61 mg/dL and calculated glomerular filtration rate of 54 mL/minute. Urinalysis showed proteinuria (192 mg/dL), with elevated protein creatinine ratio (2,526 mg/g), and microscopic hematuria (packed red blood cell [RBC]) with no RBC cast. Further biochemical analysis revealed elevated serum iron, iron saturation, and normal ferritin. Serum IgA levels were elevated at more than three times the upper limit.

**Figure 3:**
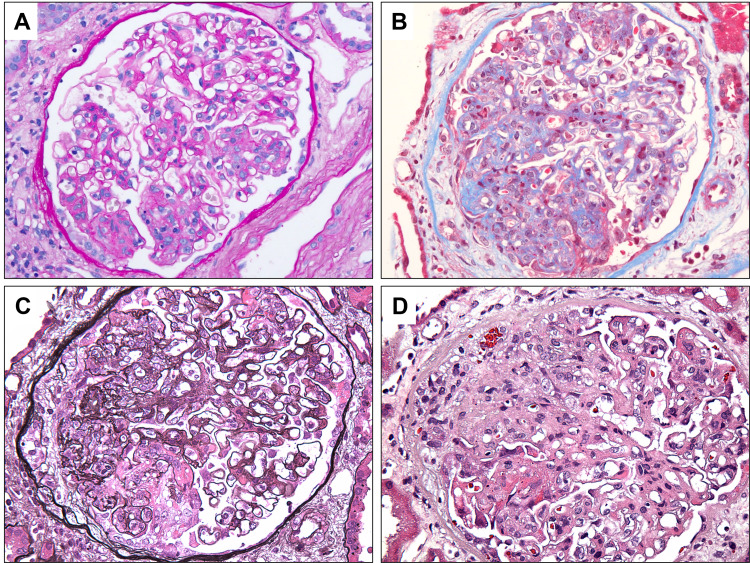
Light microscopic observation of kidney biopsy. Periodic acid–Schiff stain shows glomerulus with segmental endocapillary proliferation (A). Trichrome stain shows glomerulus with fibrocellular crescent (B). Jones’ methenamine silver stain shows endocapillary proliferation and cellular crescent (C). Hematoxylin and eosin stain shows fibrinoid necrosis and crescent (D). Original magnification 400×.

**Figure 4:**
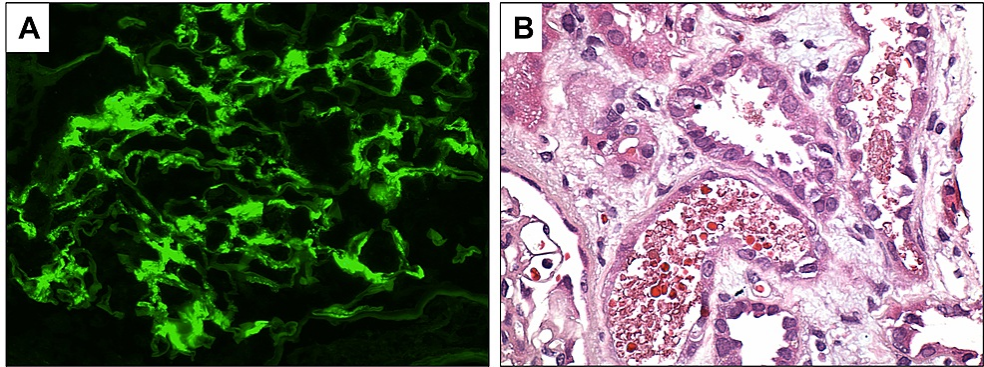
Renal biopsy of the patient. Immunofluorescence reveals mesangial deposits of immunoglobulin A (A). Hematoxylin and eosin stain shows degenerating red blood cell cast (B). Original magnification 400×.

The patient’s kidney function continued to worsen. To further evaluate the etiology and establish the diagnosis for glomerulonephritis, the patient underwent a kidney biopsy. Biopsy was significant for focal segmental endocapillary and extracapillary proliferative glomerulonephritis with focal necrotizing features (Figure 3). Immunofluorescence staining was positive for IgA deposits (Figure 4A), consistent with IgAN. Hematoxylin and eosin staining of kidney biopsy showed degenerating RBC cast (Figure 4B). Therapy was initiated with high-dose steroids and cyclophosphamide infusions. The clinical course was complicated by an upper gastrointestinal bleed from the rupture of esophageal varices for which the patient needed a transjugular intrahepatic portosystemic shunt. The patient was continued with a further course of cyclophosphamide infusions with monitoring of kidney functions.

## Discussion

In our patient, renal biopsy confirmed IgA deposit accumulation in the kidney likely secondary to alcoholic cirrhosis. Mesangial IgA deposition, which does not necessarily imply IgAN, is a frequent finding in autopsy studies in chronic liver disease [5]. However, we demonstrate an infrequent case in which our patient’s presentations including purpura, renal involvement, and abdominal pain met the latest criteria for diagnosing IgA vasculitis/HSP in adults [6]. So far, only 13 cases in addition to the presented case have reported the rare association between liver cirrhosis and HSP [7,8].

Experimentally, the relationship between excessive alcohol intake-associated hepatocellular injury and IgA nephropathy has been validated by rat models. Intense IgA deposition, mild mesangial expansion, and foot process effacement, as well as severe proteinuria, have been observed in these animal models following chronic ethanol ingestion [9,10]. Regarding patients diagnosed with cirrhosis, recent evidence also showed 67 biopsies had IgA-containing immune complex deposits within a total cohort of 118 cirrhotic patients [11]. Emerging case reports further illustrate that alcohol abuse possibly contributes to the development of secondary IgA nephropathy [12-14].

Mechanistically, alcohol induces the expression of the microsomal ethanol oxidation system (CYP2E1) and oxidative stress, resulting in increased gastrointestinal permeability and liver inflammation. The downstream effect is IgA overload caused by elevated intestinal production and reduced hepatic immune complex clearance [15].

Overall, 87% of patients with measurable IgA-containing immune complexes in serum demonstrated IgA skin deposits [16]. Of note, our patient had significantly increased serum IgA. In addition, the purpuric rash seen in our patient is considered to be a sign of IgA vasculitis, a systemic form of IgAN. However, the petechial rash can be caused by thrombocytopenia among cirrhotic patients. A skin biopsy is needed to confirm the presence of IgA deposits in blood vessels of the skin.

The treatment for cirrhosis-related IgAN focuses on the underlying liver condition; in this case, it is important to educate patients on alcohol abstinence. Several traditional immunosuppressive agents such as corticosteroids, cyclophosphamide, azathioprine, and mycophenolate mofetil have been tried to manage IgAN [17]. Furthermore, evidence has shown that the addition of angiotensin-converting enzyme inhibitors (ACEIs) or angiotensin receptor blockers (ARBs) to corticosteroids may be important in preserving proteinuric IgA nephrotic patients’ kidney function in the long-term follow-up [18-20].

## Conclusions

We described the case of an uncommon patient who presented with alcoholic liver disease associated with microscopic hematuria, proteinuria, reduced glomerular filtration rate, IgA mesangial deposits, and skin rash, identifying a secondary form of IgA vasculitis/HSP. Even though HSP in adults is rare, it is important for physicians to consider urinalysis and kidney/skin biopsy in the context of liver cirrhosis and rash, and for patients to rigorously abstain from alcohol to slow down the progression of the disease. Multiple clinical trials have provided valuable data on the benefits of kidney function regeneration with the treatment of ACEIs/ARBs.
